# Development and characterization of a prototypic pan-amyloid clearing agent – a novel murine peptide-immunoglobulin fusion

**DOI:** 10.3389/fimmu.2023.1275372

**Published:** 2023-10-02

**Authors:** James S. Foster, Manasi Balachandran, Trevor J. Hancock, Emily B. Martin, Sallie Macy, Craig Wooliver, Tina Richey, Alan Stuckey, Angela D. Williams, Joseph W. Jackson, Stephen J. Kennel, Jonathan S. Wall

**Affiliations:** Department of Medicine, University of Tennessee Graduate School of Medicine, Knoxville, TN, United States

**Keywords:** systemic amyloidosis, amyloid phagocytosis, amyloid clearance, peptide p5, peptide-antibody fusion

## Abstract

**Introduction:**

Systemic amyloidosis is a progressive disorder characterized by the extracellular deposition of amyloid fibrils and accessory proteins in visceral organs and tissues. Amyloid accumulation causes organ dysfunction and is not generally cleared by the immune system. Current treatment focuses on reducing amyloid precursor protein synthesis and slowing amyloid deposition. However, curative interventions will likely also require removal of preexisting amyloid deposits to restore organ function. Here we describe a prototypic pan-amyloid binding peptide-antibody fusion molecule (mIgp5) that enhances macrophage uptake of amyloid.

**Methods:**

The murine IgG1-IgG2a hybrid immunoglobulin with a pan amyloid-reactive peptide, p5, fused genetically to the N-terminal of the immunoglobulin light chain was synthesized in HEK293T/17 cells. The binding of the p5 peptide moiety was assayed using synthetic amyloid-like fibrils, human amyloid extracts and amyloid-laden tissues as substrates. Binding of radioiodinated mIgp5 with amyloid deposits *in vivo* was evaluated in a murine model of AA amyloidosis using small animal imaging and microautoradiography. The bioactivity of mIgp5 was assessed in complement fixation and *in vitro* phagocytosis assays in the presence of patient-derived amyloid extracts and synthetic amyloid fibrils as substrates and in the presence or absence of human serum.

**Results:**

Murine Igp5 exhibited highly potent binding to AL and ATTR amyloid extracts and diverse types of amyloid in formalin-fixed tissue sections. In the murine model of systemic AA amyloidosis, ^125^I-mIgp5 bound rapidly and specifically to amyloid deposits in all organs, including the heart, with no evidence of non-specific uptake in healthy tissues. The bioactivity of the immunoglobulin Fc domain was uncompromised in the context of mIgp5 and served as an effective opsonin. Macrophage-mediated uptake of amyloid extract and purified amyloid fibrils was enhanced by the addition of mIgp5. This effect was exaggerated in the presence of human serum coincident with deposition of complement C5b9.

**Conclusion:**

Immunostimulatory, amyloid-clearing therapeutics can be developed by incorporating pan-amyloid-reactive peptides, such as p5, as a targeting moiety. The immunologic functionality of the IgG remains intact in the context of the fusion protein. These data highlight the potential use of peptide-antibody fusions as therapeutics for all types of systemic amyloidosis.

## 1 Introduction

Systemic amyloidosis is characterized by the deposition of misfolded proteins, as amyloid fibrils, in the extracellular space of organs and tissues throughout the body (1, 2). Approximately 18 different proteins have been identified as components in systemic amyloidosis (3). The major types of systemic disease result from the misfolding and aggregation of transthyretin (ATTR amyloidosis), free monoclonal immunoglobulin light chains (AL amyloidosis) or leukocyte cell-derived chemotaxin-2 (ALECT2 amyloidosis) (4). These three types comprise more than 90% of the diagnosed cases in the US (5). Amyloid fibrils deposit in the extracellular space of organs and tissues in association with extracellular matrix components and soluble proteins sequestered from the circulation (6). Progressive amyloid deposition results in a complex matrix that, despite the presence of misfolded, non-native proteins, is not effectively cleared in most patients. This may be due to a lack of immune recognition or an inadequate immune response.

The pathologic manifestations associated with amyloidosis can be myriad, which makes early and accurate diagnosis challenging (7). The major cause of mortality is cardiac or renal failure. Cardiac amyloidosis can cause heart failure which manifests as left ventricular wall and intraventricular septal hypertrophy and functional abnormalities that result in impaired global longitudinal strain and reduced ejection volume (8). Amyloid-related renal insufficiency, a consequence of tubular and glomerular nephropathy, leads to proteinuria and worsening glomerular filtration, which ultimately results in the need for dialysis. However, these are systemic disorders (9), where amyloid can negatively impact any organ resulting in varied sequelae including impaired nerve conduction, diarrhea/constipation, shortness of breath, fatigue, and musculoskeletal issues. The correlation between amyloid load and clinical outcomes has been well established, notably based on biopsy studies and measurements of extracellular volume studies of the kidney and heart, respectively (10–13).

Current treatment strategies focus principally on reducing synthesis of the amyloidogenic precursor protein, thereby slowing amyloid formation. For patients with AL amyloidosis, this is primarily achieved with plasma cell-directed chemo- and immunotherapeutics, protease inhibitors, and autologous stem cell transplantation (14, 15). Similarly, transthyretin production by the liver is reduced using ribonucleic acid interference (RNAi) (16, 17) or ligand-conjugated antisense oligonucleotides (18). Additionally, for patients with ATTR amyloidosis, stabilization of the tetrameric transthyretin native state is achieved using small molecule ligands that prevent dissociation, which is a critical first step in amyloid formation (19–22). Despite these significant advances in the development of novel treatments for AL and ATTR amyloidosis, there are no approved methods to remove existing amyloid from organs, and prognosis often remains poor. Moreover, for patients with ALECT2 and the hereditary ultra-rare forms of amyloidosis, there are no therapeutic options. Therefore, novel reagents capable of removing amyloid present at diagnosis and reversing organ dysfunction represent the next goal in the treatment of these disorders.

Opsonization of amyloid with immunostimulatory antibodies and recruitment of macrophages capable of clearing tissue amyloid is a well-studied approach to effect amyloid clearance and potentially improve outcomes (23–25). There are currently four clinical studies underway at various stages assessing the efficacy of two AL amyloid-specific and two ATTR amyloid-specific mAbs; however, only one pan-amyloid mAb is in clinical development. Here we describe proof of concept studies that paved the way for the development of a clinical pan-amyloid clearing reagent that began with the generation of a peptide-antibody fusion prototype. The peptide, p5, is a synthetic polybasic L-amino acid reagent that is capable of binding amyloid *via* electrostatic interactions with amyloid fibrils and the associated hypersulfated heparan sulfate glycosaminoglycans (26–28). The peptide has been genetically incorporated at the N-terminal of the light chain of a hybrid murine monoclonal antibody. The antibody comprises the F(ab)2 derived from the murine (m)11-1F4 reagent (29–31) and a murine IgG2a Fc domain, a homolog of human IgG1. Herein we describe the amyloid reactivity and bioactivity of murine (m)Igp5 with respect to characteristics important to a pan-amyloid opsonin. Our findings provide support for the use of peptide-immunoglobulin fusions with pan-amyloid reactivity as a platform for the development of novel amyloid-removing therapeutics.

## 2 Methods

### 2.1 Cloning of mIgp5

The codons encoding the murine 11-1F4 mAb heavy and light chain proteins were previously sequenced in house from the Sp2/O hybridoma (clone 11-1F4). Codon-optimized cDNA encoding the 31 amino acid p5 peptide flanked by 10 and 5-amino acid spacers upstream of the 219 amino acid m11-1F4 light chain was synthesized and cloned into the pcDNA3.1_Hyg (+) vector at Genscript (Piscataway, NJ). A 20-amino acid interleukin-2 (IL-2) leader sequence was included in frame and 5’ to the peptide sequence. Similarly, optimized codons for the IL-2 secretory leader and the first 209 amino acids of the m11-1F4 heavy chain (variable heavy 1 (VH1) and constant heavy 1 (CH1)) domains) were synthesized and cloned in-frame and upstream of the hinge, CH2 and CH3 domains in a pFUSE-mIgG2A-Fc2 vector (Invivogen, San Diego, CA), at Genscript. A control light chain was also generated with lysine residues in the p5 peptide region substituted by glycine residues (designated mIgp5G). Purified plasmids were prepared at Genscript.

### 2.2 Production and purification of mIgp5 protein

mIgp5 and the negative control, mIgp5G, proteins were produced by transient transfection of HEK293T/17 cells (ATCC, Manassas, VA) using linear 25K polyethyleneimine (Polysciences, Warrington, PA) and a light to heavy chain plasmid ratio of 3:2. Cells were cultured for 9 days in DMEM/F12 (Cytiva, Logan, UT) with penicillin-streptomycin (Gibco) and 2% immunoglobulin-depleted fetal bovine serum (Cytiva). Media were changed at 3-day intervals. Transfected cell supernatants were clarified by centrifugation at 4000 x g and the secreted proteins purified by affinity chromatography using protein A-Sepharose (GE Healthcare, Pittsburg, PA) and eluted using 0.1M glycine (pH 3.0). Following overnight dialysis in PBS, products were quantified by Coomassie blue protein assay (Thermo-Pierce, Dallas, TX).

### 2.3 SDS-PAGE and Western blotting

Purified protein preparations were analyzed by gel electrophoresis using 4-12% gradient Bis-Tris polyacrylamide gels (Invitrogen) followed by staining with Coomassie brilliant blue. For Western blot analysis, proteins were transferred from the gels to nitrocellulose membranes, which were blocked with casein solution, before addition of a mouse monoclonal p5 peptide-reactive antibody (clone 13-2; in-house reagent). The membranes were then washed and incubated with horseradish peroxidase-conjugated goat anti-mouse IgG (Jackson Immunoresearch, West Grove, PA), and developed using ImmPACT diaminobenzidine peroxidase substrate (Vector Laboratories, Burlingame, CA).

### 2.4 Size exclusion chromatography

Size exclusion chromatographic analysis of mIgp5 was performed using a 7.8 x 300 mm column, 3 mm, 300 Å pore-sized matrix (Agilent Bio SEC-3: 3 mm, 300 Å). A mobile phase of PBS with 0.05% sodium azide (w/v) was used with a flow rate of 1 mL/min for 20 minutes. A 25 μL (~25 μg) sample of mIgp5 was injected, and the elution monitored by absorbance at 280 nm (to avoid interference from the sodium azide absorbance).

### 2.5 Fibrils and amyloid extracts

Amyloid-like fibrils were prepared in sterile PBS from purified rVλ6WIL (variable domain of λ6 light chain WIL), Aβ(1–40), and islet amyloid polypeptide (IAPP), as previously described (32). The fibrils were isolated from the reaction mixture by centrifugation at 15,000 × g for 5 min and resuspended in PBS. The presence of fibrils was confirmed by addition of thioflavin T solution to ~5 μg of fibril preparation and measuring the fluorescence emission at 490 nm (excitation = 450 nm). Human amyloid extracts were prepared from autopsy-derived organs using a modified water floatation method, as described elsewhere (33). Murine liver homogenates containing serum amyloid protein A amyloid (AA) were prepared as previously described (32).

### 2.6 *In vitro* binding studies—europium-linked immunosorbent assay

Binding of mIgp5 and mIgp5G to various substrates was assessed using a europium -linked immunosorbent assay (EuLISA). In the first assay, wells of a 96-well polystyrene microplate (Corning, Corning, NY, USA) were coated with poly-L-lysine (Sigma-Aldrich) followed by low molecular weight heparin (Sigma-Aldrich). Thereafter, binding of mIgp5 and m11-1F4 (Lot L0510003; provided by the National Cancer Institute) was evaluated using amyloid-like fibrils (0.83 µM), or human amyloid extracts (0.06 mg/mL), 50 μL of stock preparation per well, as the substrate. Target-coated wells were then treated with 200 µL of blocking buffer (PBS containing 1% bovine serum albumin; BSA) for 1 h at room temperature before washing with PBS and addition of the appropriate concentrations of mIgp5, mIgp5G, or m11-1F4 in PBS with 1% (w/v) BSA and 0.05% (v/v) tween 20. Following a wash step, the bound protein was detected by addition of biotinylated goat anti-mouse IgG (Jackson Immunoresearch, West Grove, PA) followed by 100 µL of europium–streptavidin (Perkin Elmer, Waltham, MA, USA) and 100 µL of enhancement solution (Perkin Elmer). Time-resolved fluorescence emission was then measured using a Wallac Victor 3 plate reader (Perkin Elmer).

For single-dose comparisons of mIgp5, mIgp5G, and m11-1F4 reactivities, patient-derived AL amyloid extracts, suspended in PBS, were coated on microplates (3µg/well), blocked with PBS containing 1% (w/v) BSA, and incubated with the test antibodies or a mIgG2a isotype control (clone MG2a-53, Biolegend, San Diego, CA) at 20nM in PBS with 1% (w/v) BSA and 0.05% (v/v) tween 20 for one hour. After washing, antibody binding was detected by incubation with biotinylated goat anti-mouse IgG Fcγ followed by europium-labeled streptavidin as described above.

### 2.7 Radioiodination of antibodies

Briefly, 100 µg of mIgp5, m11-1F4 or peptide p5 were radioiodinated with 2 mCi iodine-125 (^125^I; Perkin Elmer) using 10 µg of chloramine T as an oxidant. The radiolabeled products were purified by size-exclusion gel filtration using either; Sephadex G-25 (PD10; GE Healthcare, Pittsburgh, PA, USA) or Aca34 (Sigma-Aldrich) with a mobile phase of PBS containing 0.1% (w/v) gelatin (34). The radiochemical yield was estimated by measuring the ^125^I recovered in the purified, pooled product relative to the amount of added ^125^I. Radiochemical purity and integrity of the purified product was assessed by SDS gel electrophoresis using 10% Bis-Tris polyacrylamide gels (Invitrogen) followed by phosphor imaging (Cyclone Storage Phosphor System, Perkin Elmer, Shelton, CT, USA).

### 2.8 *In vitro* binding studies—pulldown assay

Binding of radioiodinated mIgp5, m11-1F4, or peptide p5 with amyloid-like fibrils, patient extracts or Vκ4-conjugated beads was measured, to assess differences in solution phase reactivity and as a quality control step following radioiodination, using a suspension-phase pulldown assay, as previously described (34).

### 2.9 Surface plasmon resonance measurement

Kinetic binding of mIgp5 and mIgp5G to rVλ6WIL amyloid-like fibrils was assessed using surface plasmon resonance (Reichert^®^ 2SPR; Reichert, Inc. NY, USA). Experiments were performed as described previously (32) using a carboxymethyl dextran chip derivatized with rVλ6WIL fibrils and an ethanolamine blocked channel as reference control. Sensorgrams were reported as the difference in resonance units of the rVλ6WIL fibril channel minus reference channel. Serially diluted 100 μL samples of mIgp5 or mIgp5G (starting at 40 µg/mL, ~250nM) were evaluated and the data collected for 660 s (180 s binding-phase and 480 s dissociation-phase) with a flow rate of 25 µL/min. On- and off-rate data were extracted from the sensorgram, aligned, and analyzed using the Reichert 2SPR software by fitting to the two-state binding algorithm with conformational change [A + B = AB = AB*], which provided the best fit to the kinetic data.

### 2.10 Biodistribution measurements

*In vivo* biodistribution studies of ^125^I-mIgp5 using small animal imaging, tissue radioactivity measurements and microautoradiography were performed, as previously described (35). For these studies, H2-Ld-huIL-6 Tg Balb/c mice with systemic AA amyloidosis were used. Amyloidosis was induced by intravenous injection of 100 µg amyloid-enhancing factor (AEF) (36) and were evaluated 5 wk post induction. Amyloid-free, WT mice served as controls. Mice were administered, IV in the lateral tail vein, ~100 μCi ^125^I-mIgp5 (100 µg bolus of mIgp5 with 10% (w/w) 125I-mIgp5) in a 200 µL-volume of sterile PBS containing 0.1% gelatin. Cohorts (n=4) of AA or WT mice were euthanized at 24 h, 48 h, or 72 h post-injection. Small animal single photon emission and x-ray computed tomographic (SPECT/CT) imaging was performed as previously described (37). Thereafter, the organs were harvested at necropsy for measurement of tissue radioactivity (expressed as percent injected dose per gram of tissue, %ID/g) and microautoradiographic analyses.

### 2.11 Autoradiography

Six-micrometer-thick sections were cut from formalin-fixed, paraffin-embedded blocks onto Plus microscope slides (Fisher Scientific), dipped in NTB-2 emulsion (Eastman Kodak), stored in the dark and developed after a 4-d exposure. Tissue sections were counter-stained with hematoxylin. Tissues were examined microscopically and photomicrographs were acquired using a Leica DM500 light microscope fitted with cross-polarizing filters (for Congo red). Digital microscopic images were acquired using a cooled CCD camera (SPOT; Diagnostic Instruments) and were typically acquired using a 10× objective with no digital zoom, unless otherwise noted. Consecutive tissue sections were stained with Congo Red to demonstrate the presence of amyloid.

### 2.12 Immunohistochemistry and histology

Formalin-fixed, paraffin-embedded, tissue sections were cut at 6 µM thickness and placed on Plus slides (Fisher Scientific, Norcross, GA, USA). Antigen retrieval was performed using Target Retrieval Solution™, pH 9 (Dako Corporation, Carpenteria, CA, USA), according to the manufacturer’s instructions. The tissue sections were then incubated with mIgp5 or mIgp5G at 0.15 µg/mL in PBS overnight at 4°C. The slides were then washed in water and the presence of mIgp5 visualized using avidin–biotin immunoglobulin detection kit (Elite Mouse IgG kit; Vector Laboratories, Burlingame, CA, USA) followed by development with diaminobenzidine reagent (ImmPACT™ Peroxidase Substrate kit; Vector laboratories). Tissue staining with Congo red was performed as previously described (38). Photomicrographs were acquired as described above.

### 2.13 Phagocytosis and complement activation assays

Synthetic rVλ6WIL fibrils and human amyloid extracts were labeled with the pH-sensitive fluorophore pHrodo Red succinimidyl ester (Invitrogen), according to manufacturer’s instructions. Human THP-1 monocytes (ATCC) were cultured in DMEM/F-12 (Cytiva) supplemented with 1% penicillin/streptomycin and 10% fetal bovine serum (Cytiva). THP-1 cells in 24 well tissue culture plates were differentiated into M0 macrophages by overnight incubation in 50 nM phorbol myristate acetate (PMA) (39) followed by a 3-day recovery in the absence of PMA. Differentiated surface-adherent macrophages in RPMI culture medium (Cytiva) were then co-incubated with 20 µg/well of pHrodo Red-labeled amyloid substrate for 1 h at 37°C. Uptake of amyloid substrate was quantified by capturing images of the red fluorescence in the wells using a Keyence BZ-X700E microscope (Keyence, Atlanta, GA). The images were analyzed using Image Pro Premier (Media Cybernetics) and the data expressed as the number of pixels with intensity above a pre-established threshold – positive pixels per field.

Assays for complement-mediated enhancement of phagocytosis were performed as above with the addition of up to 20% citrate-treated human plasma in the reaction well. For assays of complement activation by EuLISA, plates were coated with rVλ6WIL fibrils, incubated with mIgp5, mIgp5G control, or PBS, washed and incubated with varying concentrations of human plasma in RPMI. Deposition of active C5b9 in wells containing rVλ6WIL fibrils with mIgp5, mIgp5G or no Ab was detected using a biotinylated anti-C5b9 antibody (AbCam, Cambridge, UK) followed by detection of time-resolved fluorescence emission following addition of europium-streptavidin, as above. Wells without rVλ6WIL fibrils served as controls for non-specific activation of complement.

### 2.14 Statistical methods

Correlation analyses were performed by determining the Pearson r using a two-tailed equation with 95% confidence interval. Comparison of phagocytosis data was performed using an unpaired student t-test with α = 0.05. Biodistribution data were displayed as the mean and standard deviation (n=4). Complement activation was compared using a 2-way ANOVA with Dunnett’s correction for multiple comparisons. All analyses were performed using Prism v.9.4 (Graphpad), where ns, p>0.05, *, p ≤ 0.05, **, p ≤ 0.01, ***, p ≤ 0.001, ****, p ≤ 0.0001.

### 2.15 Ethics statement

All patient-derived tissue samples were used in accordance with an Institutional Review Board-approved application. Animal studies were approved by the University of Tennessee Institutional Animal Care and Use Committee and were performed in accordance with the guidelines provided by OLAW and the Guide for the Care and Use of Laboratory Animals. The University of Tennessee Medical Center animal program is AAALAC-i-accredited.

## 3 Results

### 3.1 mIgp5 production, characterization, and comparison of binding

**Figure 1:**
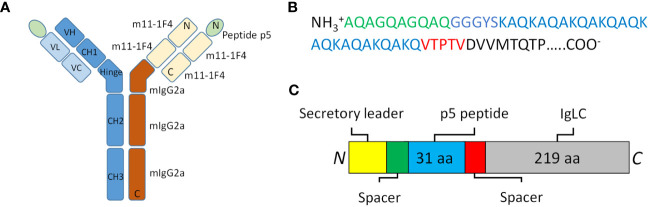
The structure of mIgp5. **(A)** Schematic representation of mIgp5 comprising the p5 peptide, murine 11-1F4 light chain variable (VL) and constant (VC) domains, and the murine IgG2a heavy chain hinge, constant two (CH2) and three (CH3) domains. **(B)** Amino acid sequence of the p5 peptide (blue) with N- and C-terminal spacers indicated (green and red), followed by the initial light chain amino acids of m11-1F4 (black). **(C)** Schematic of the full length mIgp5-light chain fusion protein structure.

**Figure 2:**
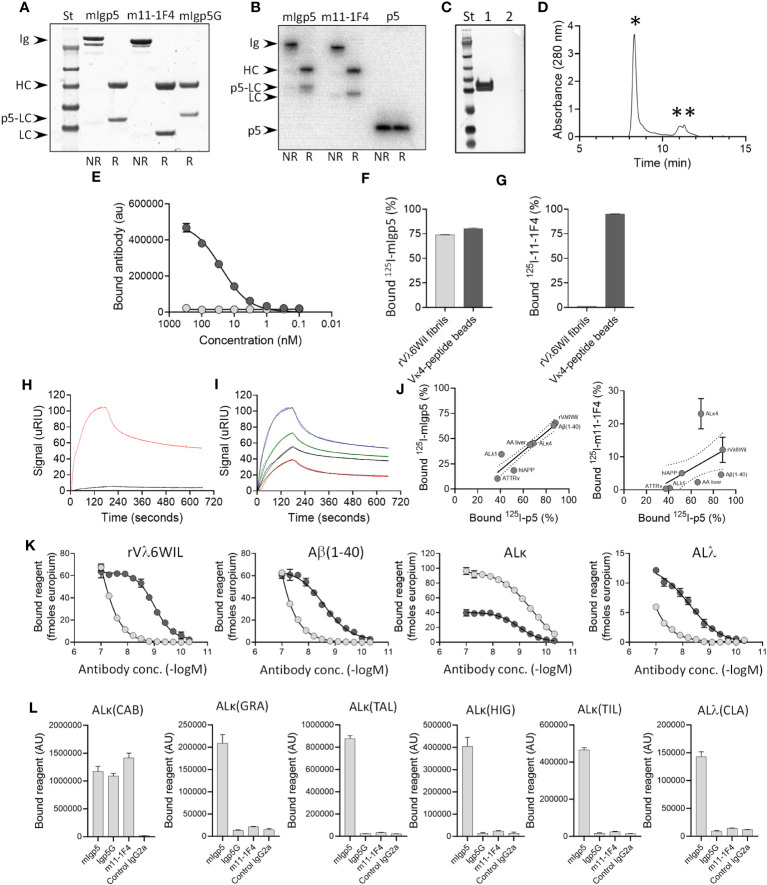
Murine Igp5 is secreted as intact immunoglobulin and binds to heparin, synthetic amyloid fibrils, and patient amyloid extracts. Structure of mIgp5, m11-1F4 and mIgp5G **(A)** and radioiodinated ^125^I-mIgp5 and ^125^I-m11-1F4 and ^125^I-p5 **(B)** assessed by SDS-PAGE under non-reduced (NR) or reduced (R) conditions where Ig, intact immunoglobulin; HC, immunoglobulin heavy chain; LC, immunoglobulin light chain; p5-LC, peptide-light chain fusion; p5, peptide p5 alone. **(C)** Western blot analysis of reduced mIgp5 (lane 1) and reduced mIgp5G (lane 2) probed with anti-p5 peptide antibody. **(D)** Size exclusion chromatography analysis of purified mIgp5 showing full length IgG (*) and low molecular weight species (**). **(E)** Binding of mIgp5 (dark grey) and mIgp5G (light grey) to heparin by EuLISA. Binding of ^125^I- mIgp5 **(F)** and ^125^I-m11-1F4 **(G)** was assessed in a pulldown assay using synthetic rVλ6WIL fibrils and Vκ4 light chain-peptide beads. Surface plasmon resonance analysis comparing mIgp5 (red) and mIgp5G (black) binding to rVλ6WIL fibrils at 250nM **(H)**, and for mIgp5 at 250 nM (blue), 125 nM (green), 62 nM (black), and 31 nM (red) **(I)**. **(J)** The correlation between ^125^I-mIgp5 (left) and ^125^I-m11-1F4 (right) with ^125^I-p5 peptide binding to human amyloid extracts (AL and ATTR), synthetic fibrils (rVλ6WIL, Aβ(1–40) and hIAPP), and liver homogenate from a mouse with AA amyloidosis in a pulldown assay. **(K)** Solid phase EuLISA binding analysis of mIgp5 (dark grey) and m11-1F4 (light grey) on rVλ6WIL fibrils, Aβ (1–40) fibrils, ALκ4 patient extract, and ALλ patient extract. **(L)** Solid phase ELISA binding of mIgp5, mIgp5G, m11-1F4 and a control mIgG2a (all at 20 nM) to varied ALκ and ALΛ human amyloid extracts. ALκ(CAB) is a κ4 amyloid.

The mIgp5 heavy and light chain constructs were prepared by cloning cDNA encoding peptide p5 and flanking amino acid linkers into, and in frame with, the amino terminus of the murine 11-1F4 kappa light chain sequence. Separately, the 11-1F4 heavy chain variable and CH1 sequences were cloned in frame with murine IgG2a hinge, CH2, and CH3 domains (**Figure 1**). Transfection of HEK293T/17 cells resulted in mIgp5 protein production and secretion into the tissue culture media. The mIgp5 and mIgp5G proteins purified by Protein A affinity chromatography migrated as distinct heavy and light chain molecules on reducing SDS-PAGE gels (**Figure 2A**). The relative migration (R*f*) of the m11-1F4 light chain was 0.60. In contrast, mIgp5 and mIgp5G derived light chains had an R*f* of 0.53 and 0.50, respectively, reflecting the addition of the peptides. The R*f* for all three heavy chain components was 0.35.

In anticipation of *in vivo* studies, purified mIgp5 was radiolabeled with ^125^I and analyzed by SDS-PAGE with phosphor-imaging of the gel. Radioiodinated m11-1F4 and peptide p5 served as controls. Radiolabeled products were observed in the gels at the appropriate molecular mass and with >90% radiopurity for each protein analyzed (**Figure 2B**). Western blot interrogation of mIgp5 using a p5 peptide-reactive antibody revealed a tight doublet banding pattern for the mIgp5 light chain (**Figure 2C** lane 1) and no reactivity with mIgp5G (**Figure 2C** lane 2). Size exclusion chromatography analysis indicated that the purified mIgp5 was principally an intact IgG (*, **Figure 2D**) with no evidence of high order aggregates, but with some low molecular weight material that was not further characterized (**, **Figure 2D**).

Peptide p5 has innate heparin binding ability. Purified mIgp5, but not the control mIgp5G, was shown, using an EuLISA, to bind heparin coated on microplate wells with high potency (**Figure 2E**). To further assess the impact of peptide p5 on binding of the mIgp5, the reactivity of radioiodinated ^125^I-m11-1F4 and ^125^I-mIgp5 with synthetic rVλ6WIL fibrils and beads coated with Vκ4-peptide (the immunogen used to generate m11-1F4) was assessed in a pulldown assay. ^125^I-mIgp5 bound both Vλ6WIL fibrils and Vκ4-peptide beads (**Figure 2F**) whereas ^125^I-m11-1F4 only bound Vκ4-peptide beads due to the absence of the peptide (**Figure 2G**). Binding kinetics of mIgp5 and mIgp5G was further assessed using surface plasmon resonance with rVλ6WIL fibrils as the substrate (**Figures 2H, I**). Using a two-state reaction model, mIgp5 binding yielded two affinity values, 4.82 nM and 435 nM (**Figure 2I**), possibly indicative of the high and low affinity binding of the peptide and F(ab)_2_, respectively. mIgp5G exhibited minimal binding to rVλ6WIL fibrils (**Figure 2H**).

To determine whether the binding of mIgp5 to varied amyloid substrates was driven by the p5 peptide or m11-1F4 F(ab)_2_, we performed a binding correlation of the reactivity of ^125^I-mIgp5 and ^125^I-m11-1F4 with that of peptide ^125^I-p5 alone in a pulldown assay (**Figure 2J**). A significant strong correlation was observed between the binding of ^125^I-p5 and ^125^I-mIgp5 (r*_p_* =0.923, *p*=0.003) indicating the reactivity of mIgp5 with amyloid is governed by peptide interactions (**Figure 2J** left). In contrast, there was no correlation between the binding of the radiolabeled peptide and ^125^I-m11-1F4 (*p*=0.269) (**Figure 2J** right).

Binding potency of mIgp5 for rVλ6WIL amyloid-like fibrils and Alκ4 or Alλ2 amyloid extracts was significantly enhanced as compared to that of m11-1F4, apart from Alκ4 amyloid (**Figure 2K**). mIgp5 exhibited saturable binding over the concentration range studied to rVλ6WIL and Aβ (1–40) fibrils, as well as Alκ4, and Alλ2 extracts, with estimated EC50 values of 1 nM (95%CI: 0.9,1.1), 2.5 nM (95%CI: 1.9,3.5), 1.1 nM (95%CI: 0.9,1.2), and 5.6 nM (95%CI: 3.7,13.9), respectively. In contrast, with the exception of Alκ4 amyloid, m11-1F4 bound with decreased potency to these substrates with estimated EC50 values of 122 nM (95%CI: 88.9,195.9), ~1.8 μM (95%CI: nd,nd), 0.3 nM (95%CI: 0.2,0.4) and ~488 μM (95%CI: nd, nd).

Using a single concentration, 20 nM, of mIgp5, mIgp5G, m11-1F4 or mIgG2a control mAb, the binding of each reagent was assessed using a panel of human AL amyloid extracts (**Figure 2L**). Binding to the ALκ(CAB), a κ4 extract, was comparable for all reagents except the control, indicating the reactivity with the 11-1F4 F(ab)_2_ remained intact in the mIgp5 and mIgp5G. In contrast, at this concentration, only mIgp5 significantly bound the other (non-κ4) AL amyloid extracts, due to the presence of the amyloid reactive p5 peptide.

### 3.2 mIgp5 retains pan-amyloid reactivity

**Figure 3:**
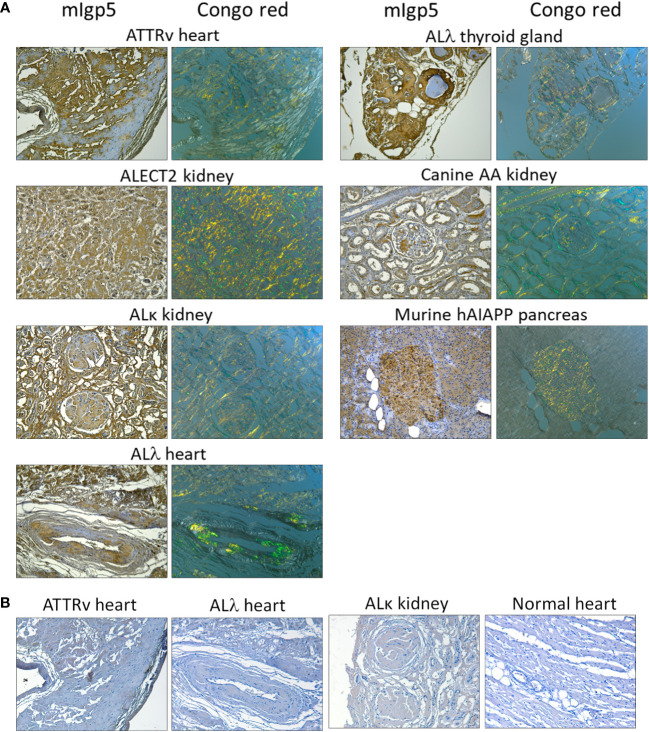
Murine Igp5 specifically binds human amyloid deposits in formalin-fixed tissues. **(A)** Immunoreactivity of mIgp5 with human ATTRv, ALλ1, ALλ3, ALκ1, and ALECT2 amyloid as well as canine AA and murine hAIAPP in formalin-fixed, paraffin-embedded tissues. mIgp5 (brown DAB staining) co-localizes with amyloid deposits seen as green-gold birefringent material in Congo red-stained consecutive tissue sections. **(B)** Murine Igp5G does not bind human ATTRv or AL amyloid in amyloid-laden tissue sections, evidenced by the lack of brown staining. No binding of the mIgp5G was seen on normal human heart indicating the lack of reactivity of the m11-1F4 component with healthy cardiac tissue. Original objective magnification 20x.

Immunostaining of amyloid *in situ*, using mIgp5, was performed using formalin-fixed paraffin-embedded tissues from patients with ATTRv, ALECT2, AL (κ or λ), as well as canine AA amyloid and human AIAPP deposits in a transgenic rat model (**Figure 3A**). In all cases, mIgp5 specifically bound tissue amyloid, as evidenced by brown diaminobenzidine staining which correlated with the distribution of amyloid, shown as green birefringent material in Congo Red-stained consecutive tissue sections. Immunostaining of cardiac ATTR and AL amyloid as well as AL renal amyloid with mIgp5G revealed no uptake in the amyloid deposits or the cardiac tissue in an amyloid-free sample (**Figure 3B**).

### 3.3 mIgp5 binds rapidly and specifically to amyloid-laden organs *in vivo*

**Figure 4:**
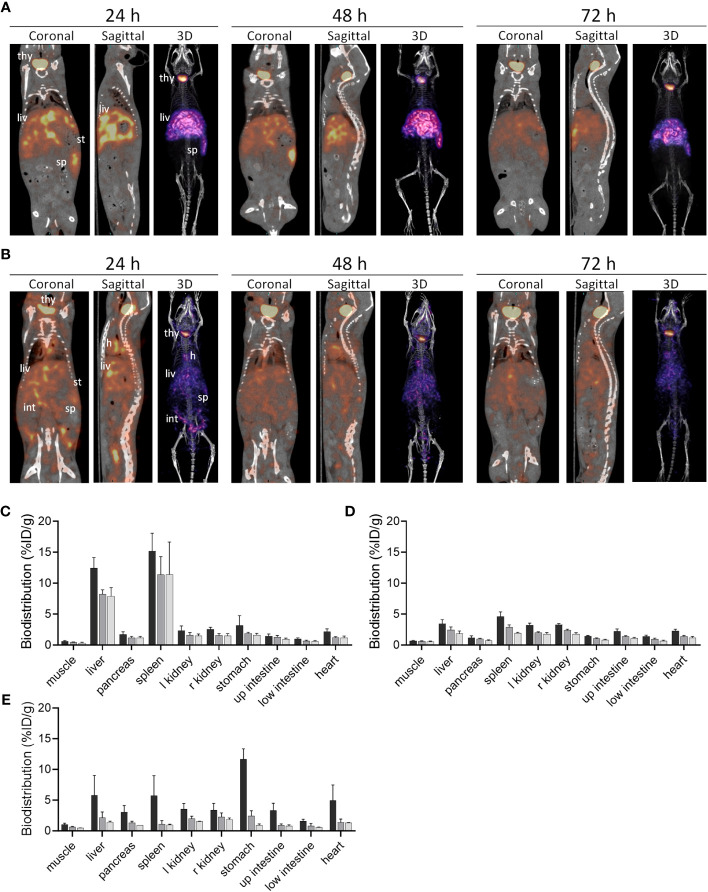
Biodistribution of ^125^I-mIgp5 in AA amyloid-laden and healthy mice. Small animal SPECT/CT imaging of mice with systemic amyloidosis **(A)** or wild type healthy animals **(B)** at 24-, 48-, and 72-hours post injection (pi). Coronal, sagittal and 3D images are shown for representative mice in the cohort (n=4), where; thy, thyroid; h, heart; liv, liver; st, stomach; int, intestines; and sp, spleen. Tissue radioactivity measurements in organs from mice with AA amyloid **(C)** and healthy animals **(D)** after receiving ^125^I-mIgp5 (black, 24 h pi; dark gray, 48 h pi; light gray, 72 h pi). The biodistribution of ^125^I-mIgp5G was similarly assessed in mice with AA amyloidosis **(E)**.

The *in vivo* biodistribution of ^125^I-mIgp5 was studied in mice with systemic AA amyloidosis and healthy wild type controls (**Figure 4**). Small animal SPECT/CT imaging of mice with AA amyloidosis at 24 h post IV injection of ^125^I-mIgp5 showed intense accumulation of radioactivity in the liver (liv) and spleen (sp), organs that contain the most amyloid in this model. Radioactivity was also observed in the unblocked thyroid gland (thy) (**Figure 4A**). Notably, the stomach (st), a site where free radioiodide transiently accumulates, was devoid of significant radioactivity (**Figure 4A** coronal image). Binding of ^125^I-mIgp5 in the liver and spleen of AA mice persisted for at least 72 h post injection (**Figure 4A**). In contrast, only diffuse radioactivity was observed in amyloid-free WT mice, associated with blood pool at all time points post injection. Radioactivity was observed in the heart (h), liver, spleen, and intestines (int) due to gastrointestinal clearance of free radioiodide that is liberated during catabolism of the antibody (**Figure 4B**). Intense radioactivity was again observed in the thyroid gland.

The retention of ^125^I-mIgp5 in organs harvested postmortem at 24 h, 48 hours, and 72 h post injection was quantified and expressed as percent injected dose per gram of tissue (%ID/g) (**Figures 4C, D**). In mice with AA amyloidosis, ^125^I-mIgp5 was observed at 7-10% ID/g in the liver and >10% ID/g in the spleen (**Figure 4C**). The quantitative retention of ^125^I-mIgp5 in these organs persisted for up to 72 h post injection, consistent with the SPECT/CT imaging findings. Radioactive accumulation in other anatomic sites was < 3%ID/g. However, in WT mice all organs had <5%ID/g at 24 h post injection (**Figure 4D**). The radioactivity in all tissues of the WT mice decreased with time consistent with excretion of the ^125^I-mIgp5. The AA : WT uptake ratio for the liver in mice with systemic AA amyloid, was 3.6 at 24 h and 4.3 at 72 h post injection, and 3.3 at 24 h and 5.8 at 72 h for the spleen.

In contrast to ^125^I-mIgp5, radioiodinated mIgp5G showed no retention in the major sites of AA amyloid deposition in the mice, the liver and spleen (**Figure 4E**). At 24 h post injection the stomach had the highest amount of radioactivity (11.6%ID/g), indicating dehalogenation of the molecule during catabolism and sequestration of the radioiodine in the stomach. The radioactivity in each tissue decreased with time and was >1.8%ID/g in all tissues sampled at 72 h post injection.

### 3.4 mIgp5 binds specifically to amyloid deposits *in vivo*

**Figure 5:**
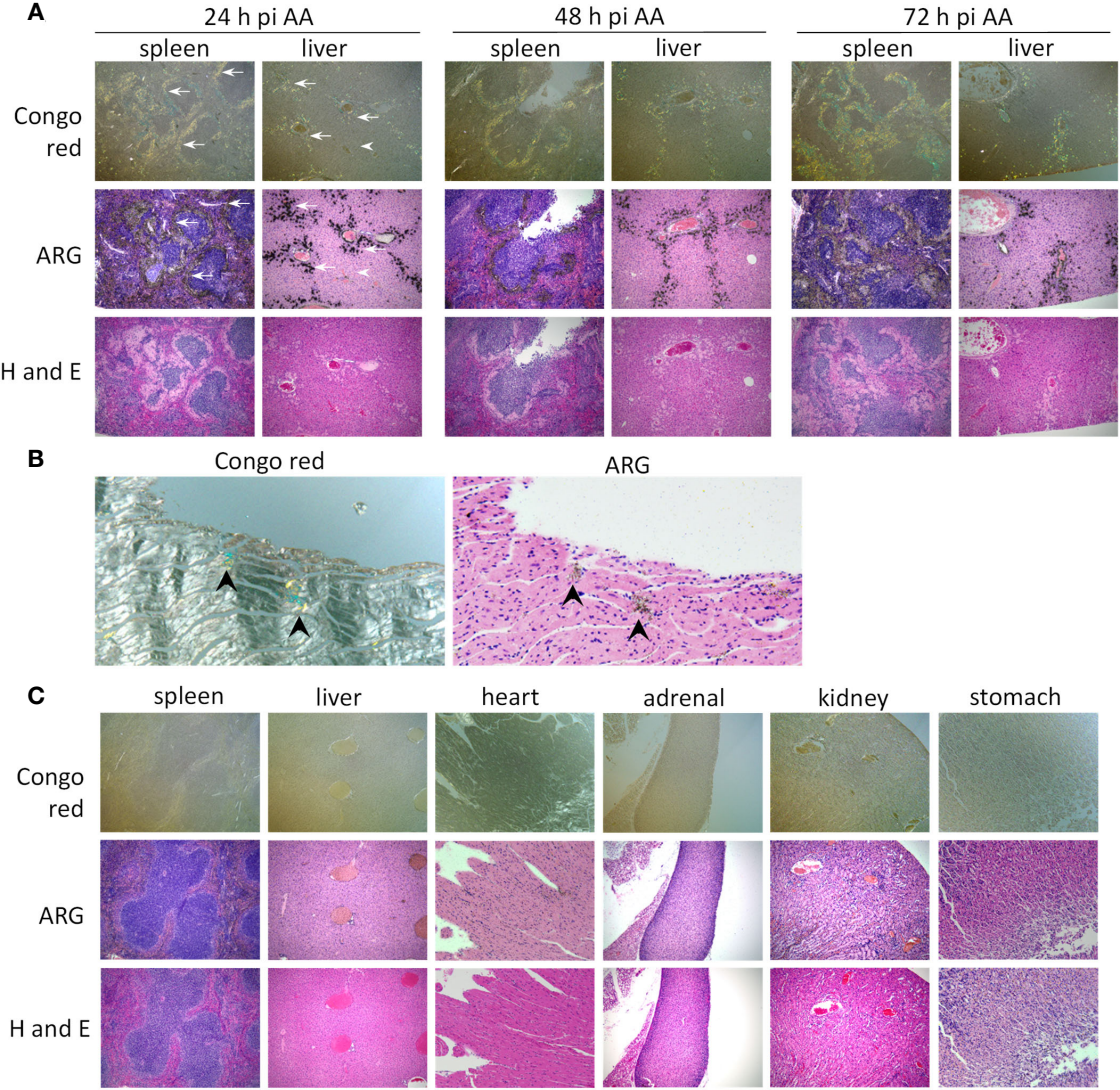
^125^I-mIgp5 specifically binds AA amyloid deposits *in vivo*. **(A)** The presence of ^125^I-mIgp5 in the spleen and liver of AA amyloid-laden mice at 24-, 48-, and 72-hours pi was evidenced by the presence of black silver deposits (arrows) in microautoradiographs (ARG) which correlated with the green-gold birefringent amyloid seen in Congo red-stained consecutive tissue sections. 10x objective magnification. **(B)** ^125^I-mIgp5 administered IV, co-localizes with Congo red-birefringent amyloid deposits in the heart tissue from AA amyloid-laden mice at 72 hours pi. 20x objective magnification. **(C)** There was no evidence in ARG of specific binding of ^125^I-mIgp5 to a range of tissues from healthy wild type mice at 24 hours pi. 10x objective magnification.

The microdistribution of ^125^I-mIgp5 in the liver, spleen and heart of these mice was assessed using microautoradiography (ARG), Congo red, and hematoxylin and eosin (H and E) staining of formalin-fixed tissue sections (**Figure 5**). In mice with AA amyloidosis, the green-gold birefringent amyloid seen in Congo red-stained sections correlated precisely with the distribution of black silver grains in the ARG stain indicative of the presence of ^125^I-mIgp5 (**Figure 5A**). The extensive perifollicular AA amyloid deposits in the spleen were visibly positive for the presence of ^125^I-mIgp5 (arrows) at all time points. Hepatic amyloid filling the sinusoids, appearing as spherical deposits, exhibited intense ^125^I-mIgp5 uptake in the ARG (**Figure 5A** arrows). Amyloid-free hepatic tissue contained little or no visible radiolabeled antibody (arrowhead). Cardiac amyloid deposition in this mouse model is not pronounced; however, scant deposits occur at the base of the left ventricle. At 72 h post injection, retention of ^125^I-mIgp5 in cardiac AA amyloid was readily appreciated in small Congo red-positive deposits in the left ventricular wall (**Figure 5B** arrowheads).

In contrast, at the earliest time point, 24 h post injection, no intense or specific uptake of ^125^I-mIgp5 was observed in spleen, liver, heart, adrenal glands, kidney, or stomach wall of amyloid-free WT mice (**Figure 5C**). Instead, diffuse radioactivity was observed, notably throughout the hepatic sinusoids, indicative of mIgp5 in the blood pool consistent with the SPECT/CT findings (**Figure 4B**).

### 3.5 mIgp5 is an effective amyloid opsonin and complement activator

**Figure 6:**
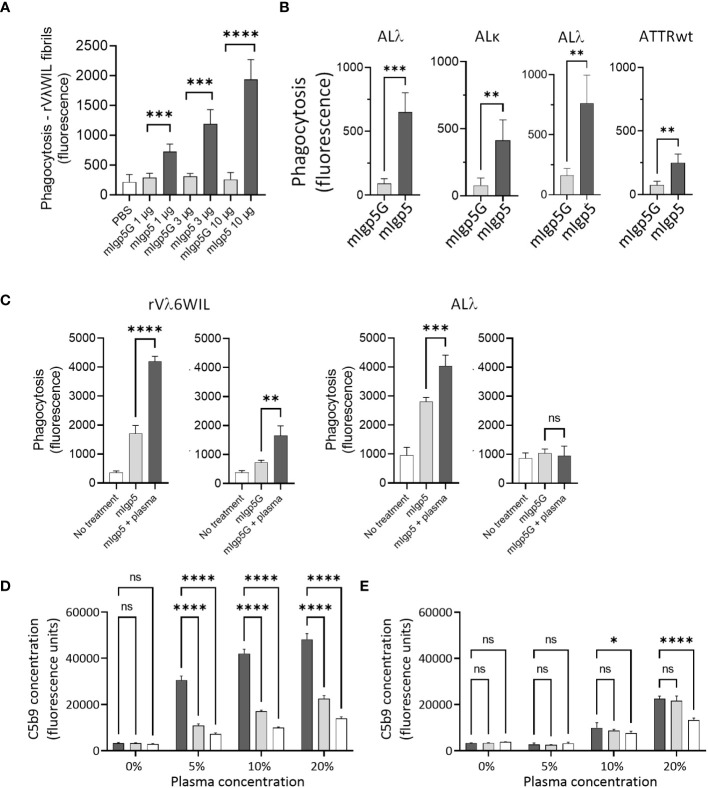
Murine Igp5 serves as an opsonin promoting phagocytic amyloid uptake and activates complement. *In vitro* phagocytosis of pHrodo red-labeled rVλ6WIL fibrils **(A)** or patient-derived amyloid extracts **(B)** by activated human THP-1-derived M0 macrophages was significantly greater in the presence of mIgp5 than with control mIgp5G. **(C)** Addition of 10% human plasma further enhanced phagocytosis of the fibrils (left) and amyloid extract (right). Complement activation (C5b9 detection by EuLISA), by mIgp5 (dark bars), mIgp5G (light grey bars), or no opsonin (clear bars) in the presence **(D)** or absence **(E)** of rVλ6WIL fibrils at increasing plasma concentrations. p>0.05, *, p ≤ 0.05, **, p ≤ 0.01, ***, p ≤ 0.001, ****, p ≤ 0.0001.

*Ex-vivo* phagocytosis assays were performed using rVλ6WIL fibrils and patient-derived amyloid extracts labeled with the pH sensitive fluorophore pHrodo Red. Human THP-1 cells were stimulated to M0 macrophage-like cells and incubated with pHrodo Red labeled fibrils or extracts in the presence of mIgp5 or the control mIgp5G antibody (**Figure 6**). In the presence of mIgp5, there was a dose-dependent increase in phagocytosis of rVλ6WIL fibrils by macrophages, while mIgp5G had no effect (**Figure 6A**). Murine Igp5 (10 μg) also induced phagocytosis of patient-derived AL and ATTR amyloid extracts significantly more effectively as compared to mIgp5G (**Figure 6B**). To evaluate whether complement could influence mIgp5-mediated phagocytosis of rVλ6WIL fibrils and an AL amyloid extract, human plasma was used as a source of complement factors (**Figure 6C**). Addition of 10% human plasma, in addition to mIgp5, resulted in a significant increase in phagocytosis of both synthetic fibrils (**Figure 6C** left) and AL amyloid extract (**Figure 6C** right). Addition of plasma enhanced the non-specific uptake of rVλ6WIL fibrils by mIgp5G but had no impact on the uptake of amyloid extract. Overall mIgp5 induced a significantly greater increase in phagocytosis of both substrates as compared to mIgp5G. Activation of complement was confirmed using a complement C5b9 detection EuLISA (**Figures 6D, E**). Increasing concentrations of human plasma were added to rVλ6WIL fibrils in the presence of mIgp5 (dark), mIgp5G (light) or no opsonin (white). Opsonization by mIgp5 resulted in a dose dependent increase in C5b9 deposition that was significantly higher at each concentration than the controls (**Figure 6D**). In the absence of fibrils, non-specific production of C5b9 by mIgp5 (dark), mIgp5G (light) or no opsonin (white) was evidenced only at higher concentrations of plasma (**Figure 6E**).

## 4 Discussion

The deposition of amyloid in tissues and organs results in significant morbidity, negatively impacts quality of life and is the source of mortality for patients with systemic amyloidosis. In recent years, effective treatment options, including stabilizers and genetic silencers for patients with ATTR amyloidosis and plasma cell immunotherapeutics for those with AL amyloidosis, have significantly improved patient outcomes. However, the clinical impact of preexisting amyloid deposits in the heart, kidneys, gastrointestinal tract, and nerves remains significant, and treatment options to clear them could prove transformative and potentially curative. Current approaches to effect amyloid removal rely on engaging the innate immune system, notably phagocytic macrophages, following opsonization by amyloid-reactive monoclonal antibodies (mAbs) or immunostimulatory fragments thereof (40, 41).

Most amyloid-reactive antibodies are specific for a single type of disorder (42). Two mAbs that were serendipitously shown to bind AL amyloid deposits are in clinical development (24, 43). Both the humanized birtamimab (NEOD001 or murine 2A4) (44, 45) and the chimeric anselamimab (CAEL-101 or murine 11-1F4) (35, 46, 47) bind cryptic epitopes present on light chain amyloid fibrils but not light chains in their native structural state, thereby enabling fibril-specific reactivity (30, 31) Birtamimab is now in a pivotal Phase 3 evaluation after demonstrating significant benefit in all cause mortality in those patients with Mayo Stage IV cardiac amyloidosis (45). Encouraging cardiorenal signals were seen in 63% (15/24) of patients in the open label Phase 1 a/b trial of the chimeric CAEL-101 (48) despite the historic observation of only 50% (9/18) patient-specific sensitivity in the uptake of iodine-124-labeled m11-1F4, by PET/CT imaging, where no uptake was seen in cardiac or renal amyloid (49).

Two TTR-binding mAbs are also being assessed in the clinic. Naturally occurring mAbs that recognize amyloid have been identified in clinical preparations of pooled immunoglobulin from healthy donors (50). Furthermore, naturally occurring ATTR amyloid-reactive antibodies have been identified in a small proportion of patients (0.002%, n=3/1663) in whom complete clearance of amyloid and restoration of normal cardiac function has been documented (51). The mAb NI006 (25) is a naturally occurring TTR-reactive mAb that has been developed for clinical use. Recent data from the early-stage evaluation of NI006 in patients with ATTRv and ATTRwt indicated decreases in cardiac extracellular volume and serum NT-proBNP levels during a 12-month study (52). This suggests that amyloid-reactive antibodies can access cardiac amyloid and potentially effect clearance. The second TTR-reactive mAb, NNC6019 (PRX004) binds a cryptic epitope concealed in the interface of the native tetramer but exposed on monomeric TTR and in the fibrillar form (53). Early clinical data on therapeutic efficacy of NNC6019 suggest positive effects on cardiac amyloid, with improvement in global longitudinal strain, and reduction in the concentration of circulating non-native TTR (52).

The m11-1F4 F(ab)_2_ was used in the mIgp5 construct since it has inherent AL amyloid fibril reactivity, and molecular imaging in mice and patients with AL amyloidosis indicated no significant off-target reactivity with healthy tissues (49, 54). Addition of the pan amyloid peptide p5 at the N-terminal of the m11-1F4 light chains enhanced the binding potency of mIgp5 for AL amyloid substrates relative to m11-1F4 and expanded the reactivity to other types of amyloid (**Figure 2**). Moreover, the p5 peptides bind two ubiquitous components of all amyloid deposits, fibrils and hypersulfated heparan sulfate, which may increase the binding density on amyloid with positive down-stream effects on phagocytosis due to enhanced FcR cross-linking and cellular activation (55). Two pan-amyloid antibodies have been developed and assessed clinically. The first, dezamizumab, bound the ubiquitous amyloid-associated serum amyloid P (SAP). Due to the presence of SAP in the circulation, use of dezamizumab required pre-treatment with miridesap ((^®^-1-{6-[(R)-2-carboxypyrrolidin-1-yl]-6-oxohexanoyl}pyrrolidine-2-carboxylic acid)), which clears SAP from the circulation (56). In the Phase 1study, a small number of patients (~30%) exhibited reduced amyloid burden, notably in the liver, based on reduced ^123^I-SAP organ uptake by planar gamma scintigraphic imaging. Although the ^123^I-SAP imaging data may be complicated by the fact that the treatment used anti-SAP antibodies, this observation suggested that amyloid-bound mAbs could induce meaningful clearance of amyloid deposits in abdominal organs. Clinical development of dezamizumab was halted due to lack of efficacy in cardiac amyloidosis and adverse events possibly associated with binding to residual serum SAP (57). The second pan-amyloid therapeutic, AT-02, is a newly developed humanized and optimized derivative of mIgp5 that recently entered clinical evaluation (NCT05951049). Based on microautoradiographic data obtained with mIgp5 (**Figure 5**), we anticipate no discernible binding to healthy tissues. We therefore anticipate that AT-02 will exhibit specific and potent amyloid binding *in vivo*.

Murine Igp5 is the third immunotherapeutic that utilizes pan-amyloid reactive peptides as the amyloid targeting moiety. Initially, an Fc-peptide fusion was developed that bound amyloid of diverse types and colocalized with amyloid in a murine model of systemic amyloidosis (41). In parallel, a two-step treatment strategy was envisaged using a p5 peptide-epitope fusion (peptope) to pretreat the amyloid before administering an opsonizing IgG1 mAb with high affinity for the epitope on the peptope fusion (35). The current mIgp5 construct, is a murine IgG1-IgG2a hybrid immunoglobulin with the peptide fused genetically to the N-terminal of the immunoglobulin light chain and represents a further development of immunotherapeutics utilizing these peptides. Coincident with therapy development, an analogous peptide, p5 + 14, radiolabeled with the positron-emitting radionuclide iodine-124 (iodine-124-evuzamitide) has been evaluated for detecting amyloid in PET/CT and PET/MR imaging studies of patients with systemic amyloidosis and healthy subjects (e.g. NCT03678259 and NCT05758493). This peptide, structurally identical to p5 but with a 14 amino acid extension and +12 net charge, rapidly bound diverse types of amyloid throughout the body (58–60) with high sensitivity, notably in the heart, supporting the hypothesis that the p5 peptide, in the context of mIgp5, drives the specific, high potency reactivity of the fusion with multiple types of amyloid.

Murine Igp5 is designed to serve as an opsonin to recruit and stimulate macrophages for amyloid clearance. Tissue resident macrophages are present in most organs, including the heart (61), but amyloid is not recognized as foreign by these cells, despite the presence of misfolded (non-native) proteins in the fibrils. This may be due to protective effects of extracellular matrix proteins, such as collagen, in the amyloid deposits, which has been shown to inhibit the uptake of amyloid-like fibrils by macrophages *in vitro* (62). Amyloid-reactive mAbs can be used as opsonins to overcome this immunologic apathy by the engagement of macrophages *via* Fc-receptor interactions, the promotion of phagocytosis, and the clearance of pathologic deposits. In humans, this is most effectively achieved using the IgG1 isotype; however, in mice, the IgG2a used in mIgp5 is most effective (63, 64). Amyloid-bound mIgp5 effectively fixed complement *in vitro* as evidenced by production of the terminal C5b9 complex (**Figure 6D**).

Characterization of mIgp5 produced by transient expression of HEK293T/17 cells indicated that the Ig light chain protein has a higher molecular weight as compared to the parental m11-1F4 light chain, due to the presence of the p5 peptide. However, a light chain doublet was observed in the western blot analysis, which suggests that the p5 peptide is not entirely intact in the preparations evaluated (**Figure 1B**). This may result from erroneous proteolytic cleavage of the peptide N-terminal during removal of the upstream signal peptide, or from the action of intracellular proteases released during the 9-day fed batch culture, despite the presence of 2% fetal bovine serum. In an optimized clinical candidate, strategies to generate an Ig-peptide fusion could be employed to minimize peptide cleavage and ensure a homogeneous preparation with intact amyloid-reactive peptide. This would enhance amyloid binding potency, which is the first critical property of an effective opsonin.

Systemic amyloidosis results from the misfolding and aggregation of proteins with diverse primary and tertiary structures. Therefore, generating a mAb immunotherapeutic for each type of amyloid with specificity for the fibril and not the precursor protein is challenging, and likely cost prohibitive. Thus, the production of reagents with pan-amyloid reactivity is clinically important, particularly for the underserved patients with rarer types. Murine Igp5 is a prototypic reagent that uses the pattern recognition peptide p5 to facilitate pan amyloid reactivity and, like the radioiodinated p5 + 14 peptide imaging agent, a clinical derivative of this prototype could be clinically transformative for all patients with systemic amyloidosis. The binding and immunological characteristics of this reagent demonstrate the potential of IgG-peptide fusions and support the development and clinical evaluation of an optimized human variant. Furthermore, the potential use of peptide p5, and similar variants, to ferry immunostimulatory biologicals to amyloid could be further explored.

## Data availability statement

The raw data supporting the conclusions of this article will be made available by the authors, without undue reservation.

## Ethics statement

The studies involving humans were approved by University of Tennessee Graduate School of Medicine Institutional Review Board. The studies were conducted in accordance with the local legislation and institutional requirements. Written informed consent for participation was not required from the participants or the participants’ legal guardians/next of kin because Archived samples of human amyloid extract used in this study were isolated from samples obtained at autopsy from approximately 1990 – 2010. Samples were obtained post-mortem. The animal study was approved by University of Tennessee Institutional Animal Care and Use Committee. The study was conducted in accordance with the local legislation and institutional requirements.

## Author contributions

JF: Conceptualization, Investigation, Methodology, Validation, Writing – review & editing. MB: Investigation, Methodology, Writing – review & editing. TH: Investigation, Methodology, Writing – review & editing. EM: Writing – review & editing. SM: Investigation, Methodology, Writing – review & editing. CW: Investigation, Methodology, Writing – review & editing. TR: Investigation, Methodology, Writing – review & editing. AS: Investigation, Methodology, Writing – review & editing. AW: Investigation, Methodology, Writing – review & editing. JJ: Investigation, Writing – original draft, Writing – review & editing, Data curation, Formal Analysis, Project administration. SK: Investigation, Writing – original draft, Writing – review & editing, Conceptualization, Methodology. JW: Conceptualization, Data curation, Funding acquisition, Resources, Supervision, Writing – original draft, Writing – review & editing.
